# Supplementary material for: eQTLs identify regulatory networks and drivers of variation in the individual response to sepsis
Source: Cell Genom. 2024 Jun 18;4(7):100587. doi: 10.1016/j.xgen.2024.100587 (PMC11293594; doi:10.1016/j.xgen.2024.100587)
Supplement: Document S1. Figures S1‒S20 and Tables S1 and S2 [file mmc1.pdf]

Cell Genomics, Volume *4*

Supplemental information

eQTLs identify regulatory networks and drivers of variation in the individual response to sepsis

Katie L. Burnham, Nikhil Milind, Wanseon Lee, Andrew J. Kwok, Kiki Cano-Gamez, Yuxin Mi, Cyndi G. Geoghegan, Ping Zhang, GAinS Investigators, Stuart McKechnie, Nicole Soranzo, Charles J. Hinds, Julian C. Knight, and Emma E. Davenport

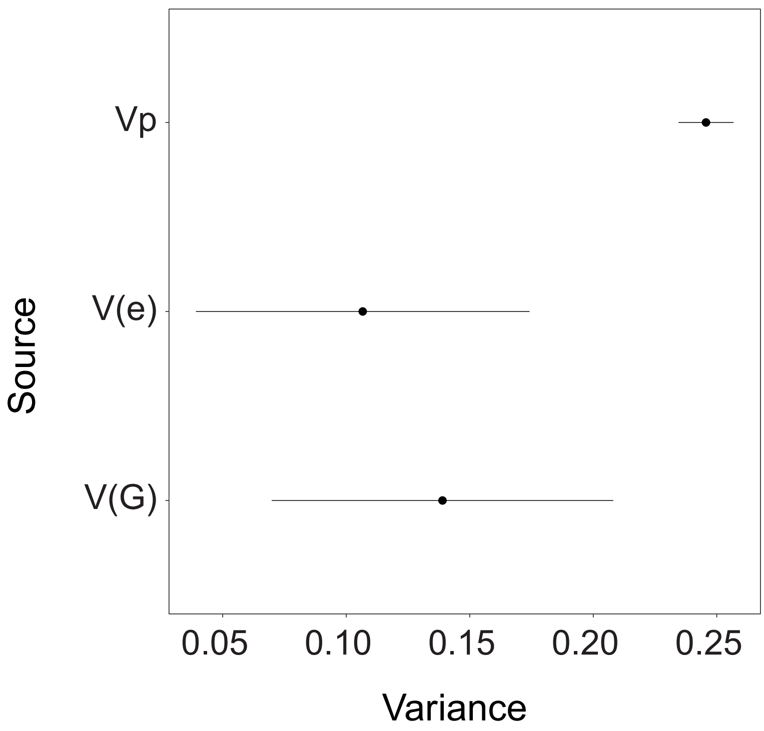

V(G)/Vp=0.57 (0.29−0.84)   p=0.019

Figure S1: Genetic contribution to the SRS1 phenotype, related to Figure 1.
The contribution of common SNPs to variation in SRS status, summarised for each patient as “Ever assigned to SRS1 in the first 5 days in ICU” vs “Never assigned to SRS1”, was estimated using GCTA. Estimates of variance in the phenotype (Vp), the variance explained by environmental factors (V(e)) and by common SNPs (V(G)) are shown as a forest plot. The proportion of the phenotypic variance explained by common SNPs collectively and the p-value calculated from likelihood ratio test are stated underneath.

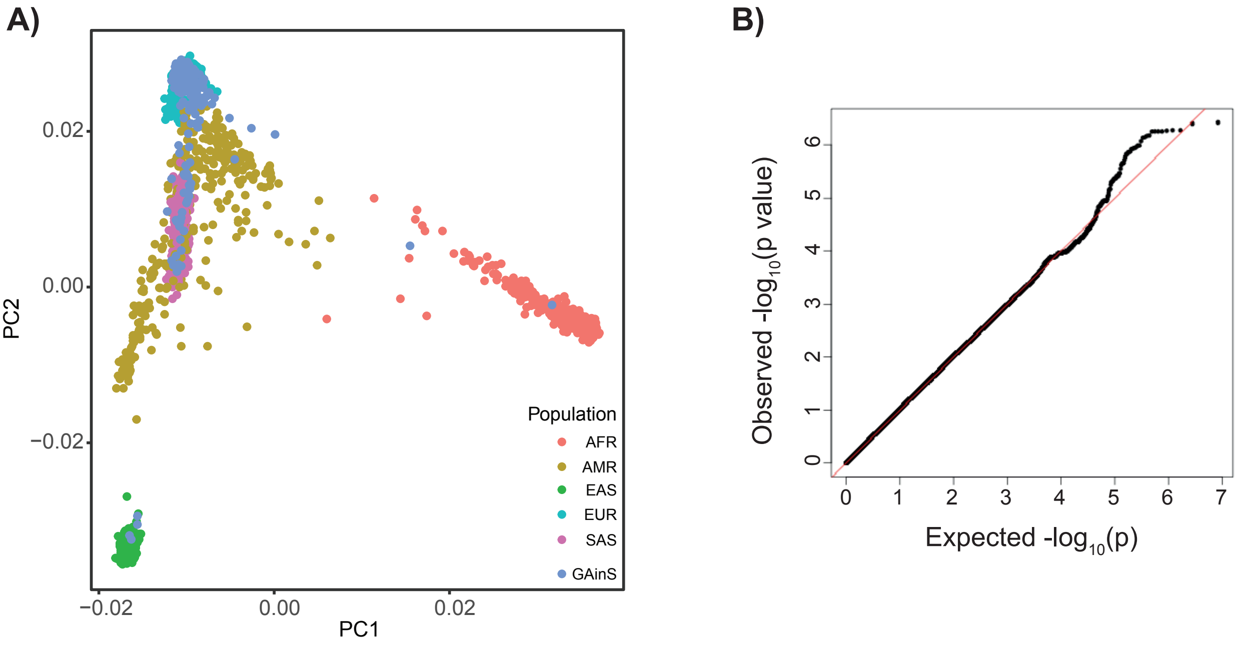

Figure S2: Genome-wide association study for SRS1ever vs SRS1never, related to Figure 1.

A) Samples were projected into the principal component space of samples from the 1000 Genomes Project.

B) Common SNPs (MAF>1%) were tested for association with the SRS1ever vs never phenotype by logistic regression. qqplot showing the observed p-value distribution plotted against the expected distribution.

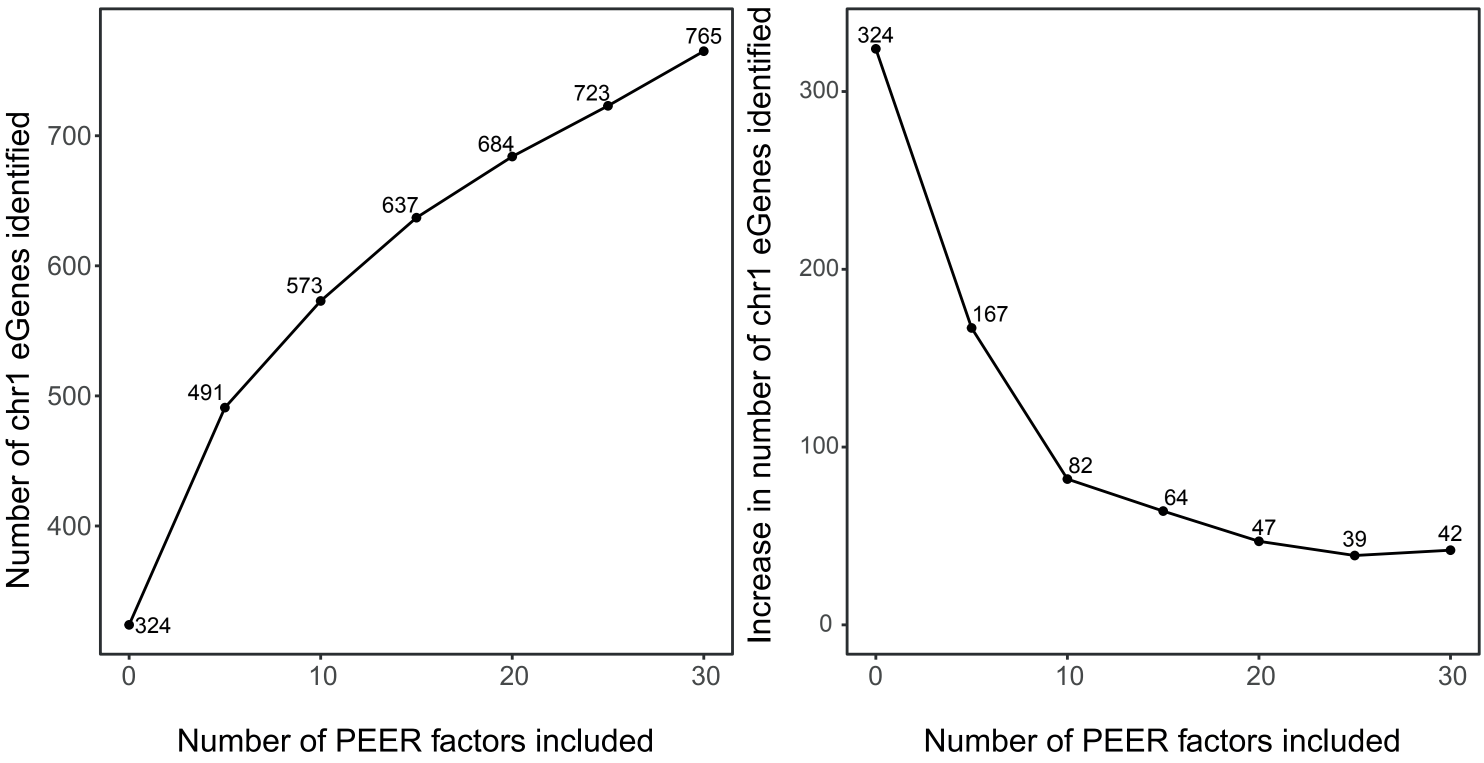

Figure S3: Impact of the number of PEER factors included in the eQTL model, related to Figure 1.
As more PEER factors are included in the eQTL model, the number of eQTLs detected on chr1 increases non-linearly (*left*). The number of additional eQTLs detected with every additional 5 PEER factors decreases rapidly (*right*).

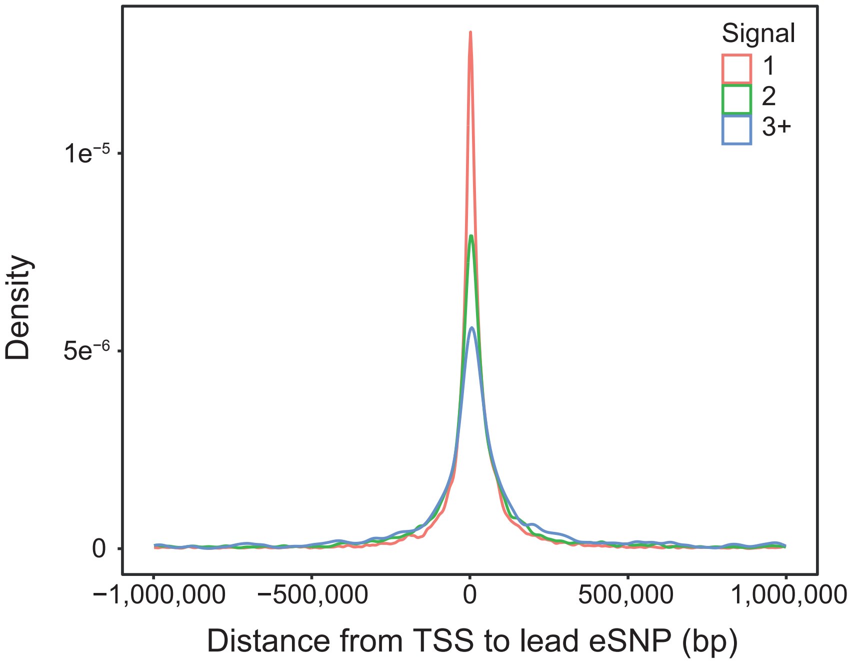

Figure S4: Distribution of conditional lead eQTL SNPs around the eGene transcriptional start site (TSS), related to Figure 1.
The base pair distance from the eSNP to its eGene's TSS was calculated and plotted as density plots by the rank of the conditional eQTL signal. Primary signals (red) were closer to the TSS than secondary signals (green), which were in turn closer than tertiary and greater signals (blue).

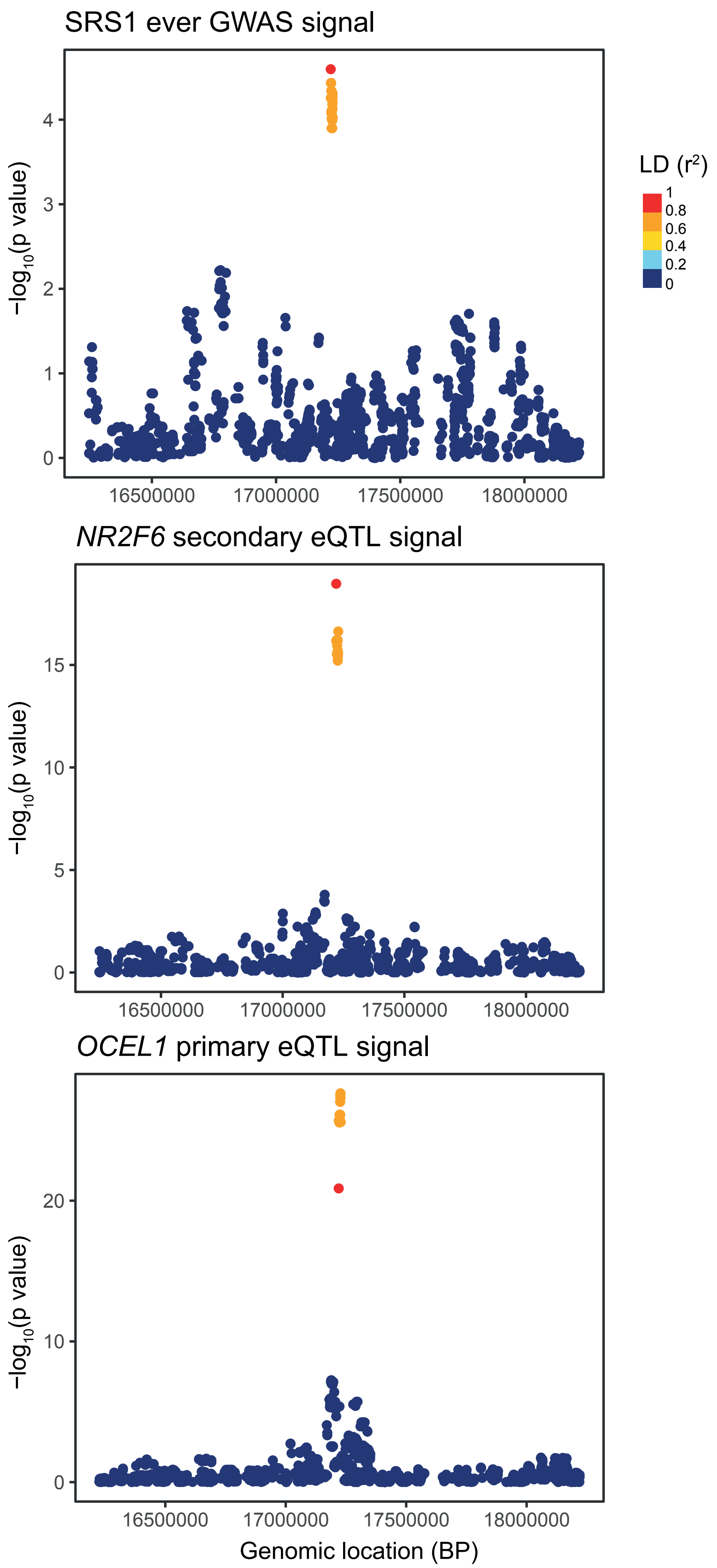

Figure S5: Colocalisation of an SRS GWAS signal with eQTL signals for *NR2F6* and *OCEL1*, related to Figure 1.
A SNP that passed the genome-wide suggestive threshold in the SRS GWAS was also a significant eQTL for *NR2F6* and *OCEL1*, and testing for colocalisation with coloc indicated that the same causal SNP was driving the three signals. Each point is a variant, with significance for each association from logistic regression (GWAS) and linear mixed model (eQTL) plotted against genomic location and colour indicating LD (r^2) with the lead SNP from the GWAS association.

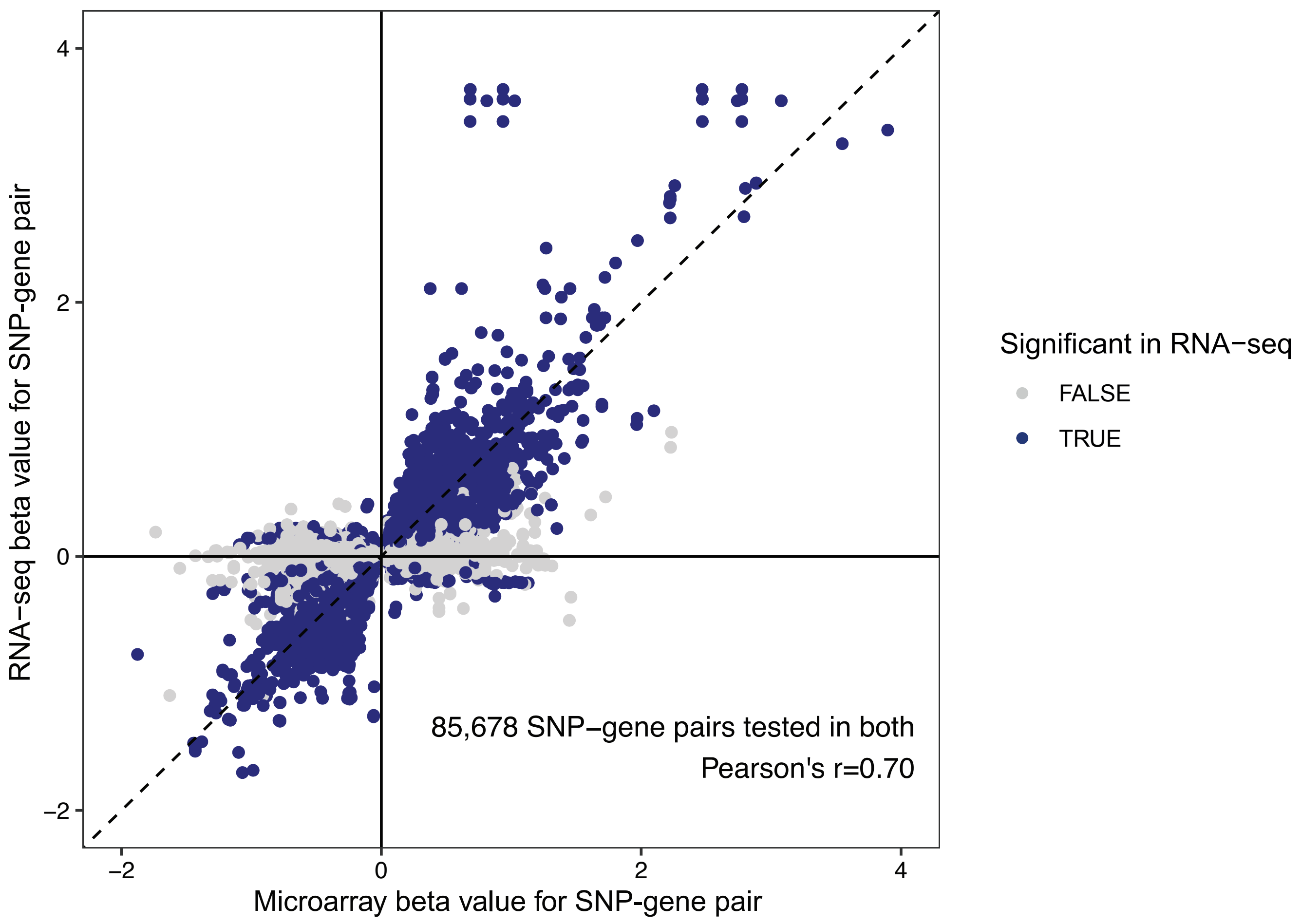

Figure S6: Replication of eQTL results from a microarray sepsis cohort, related to Figure 1.
Comparison of beta values for all SNP-gene pairs with nominal significance in our previous microarray eQTL study that were also tested in this RNA-seq cohort. RNA-seq statistics calculated using the subset of non-overlapping samples. Blue colour indicates significance in the current study. Correlation estimated by Pearson's r.

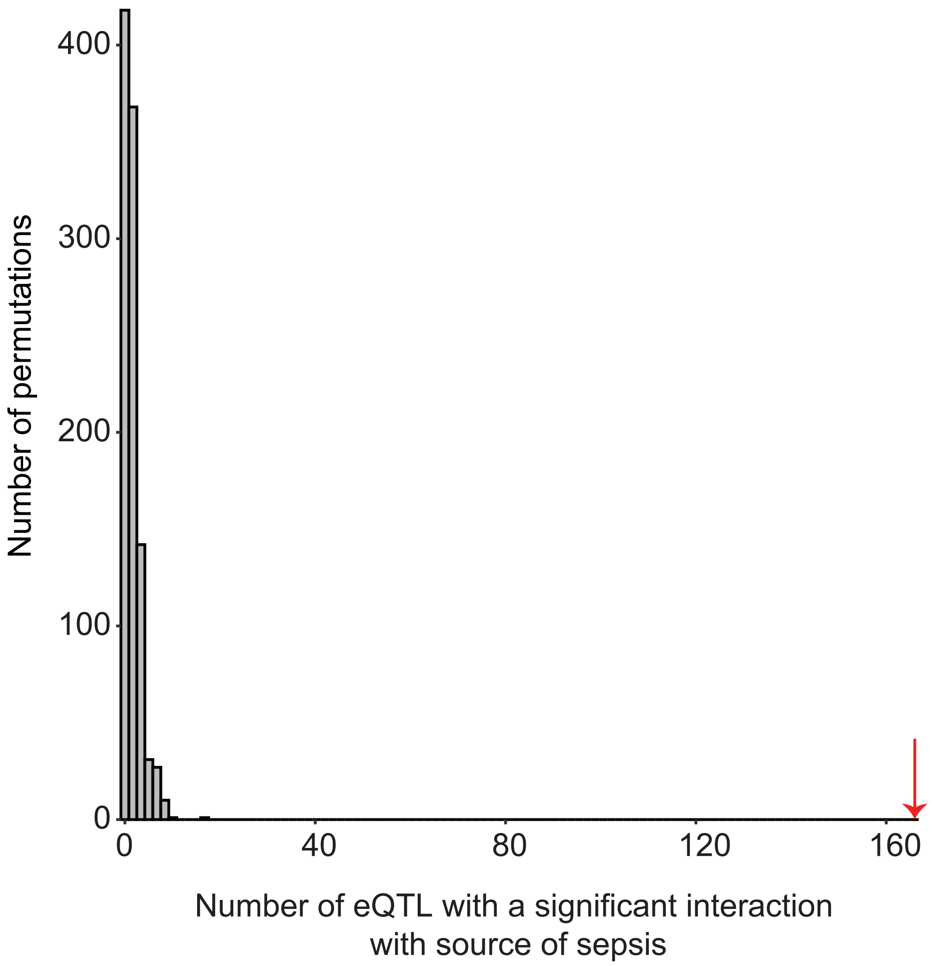

Figure S7: Distribution of source of sepsis interaction QTL from permutation analysis, related to Figure 1.
Source of sepsis was permuted across individuals and the eQTL interaction analysis was repeated, with the number of significant interactions for each permutation plotted as a histogram. The observed result is marked with a red arrow.

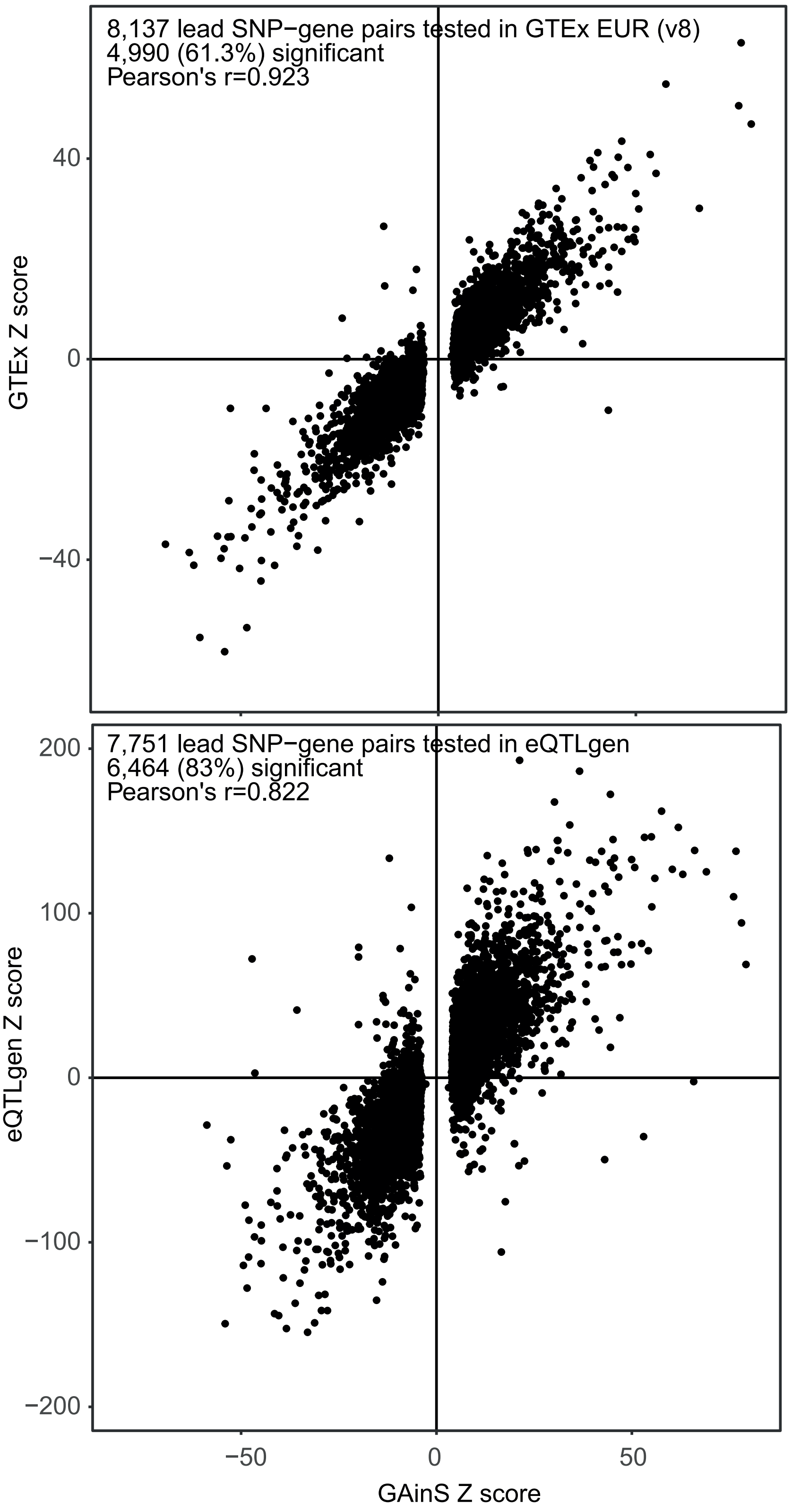

Figure S8: Comparison of eQTLs significant in sepsis to GTEx and eQTLgen results, related to Figure 1.

Comparison of z-scores for lead SNP-eGene pairs significant in sepsis and GTEx version 8 whole blood eQTL results from European individuals (top) and eQTLGen (bottom). Significance is as defined by GTEx and eQTLGen and correlation calculated with Pearson's r.

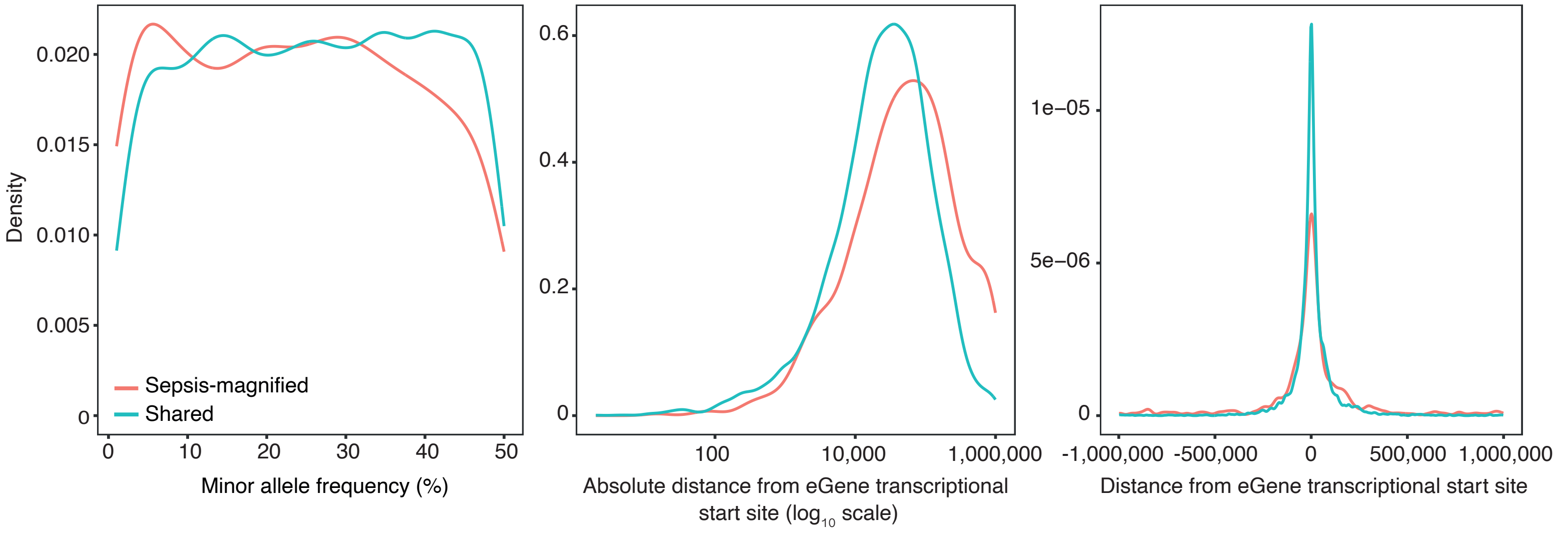

Figure S9: Characteristics of sepsis-magnified eQTL variants, related to Figure 1.
Density plots demonstrating how eSNPs involved in sepsis-magnified eQTLs differ from eSNPs involved in eQTLs with comparable effect sizes to GTEx in terms of (left) MAF, (middle and right) distance to the transcriptional start site (TSS) of the eGene. Mann-Whitney test p-values for MAF p=0.00015, |distance| p=2.5e-27

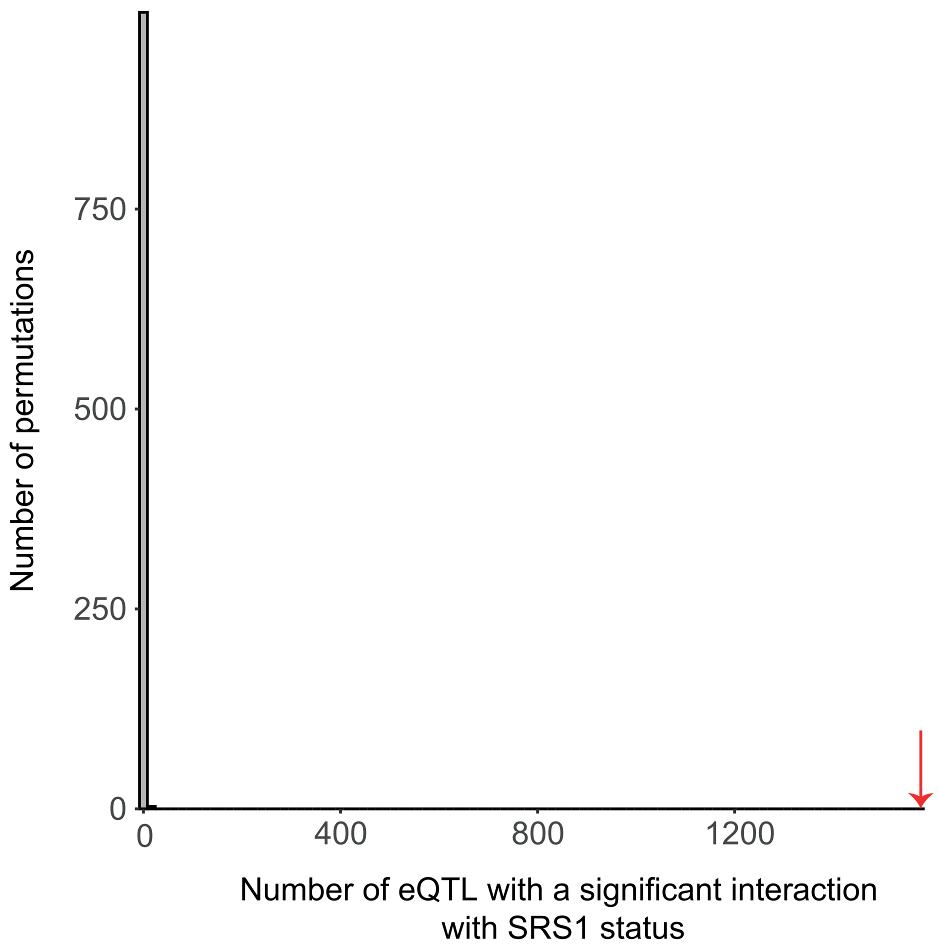

Figure S10: Distribution of SRS interaction QTL from permutation analysis, related to Figure 2.
SRS status was permuted across samples and the eQTL interaction analysis was repeated, with the number of significant interactions for each permutation plotted as a histogram. The observed result is marked with a red arrow.

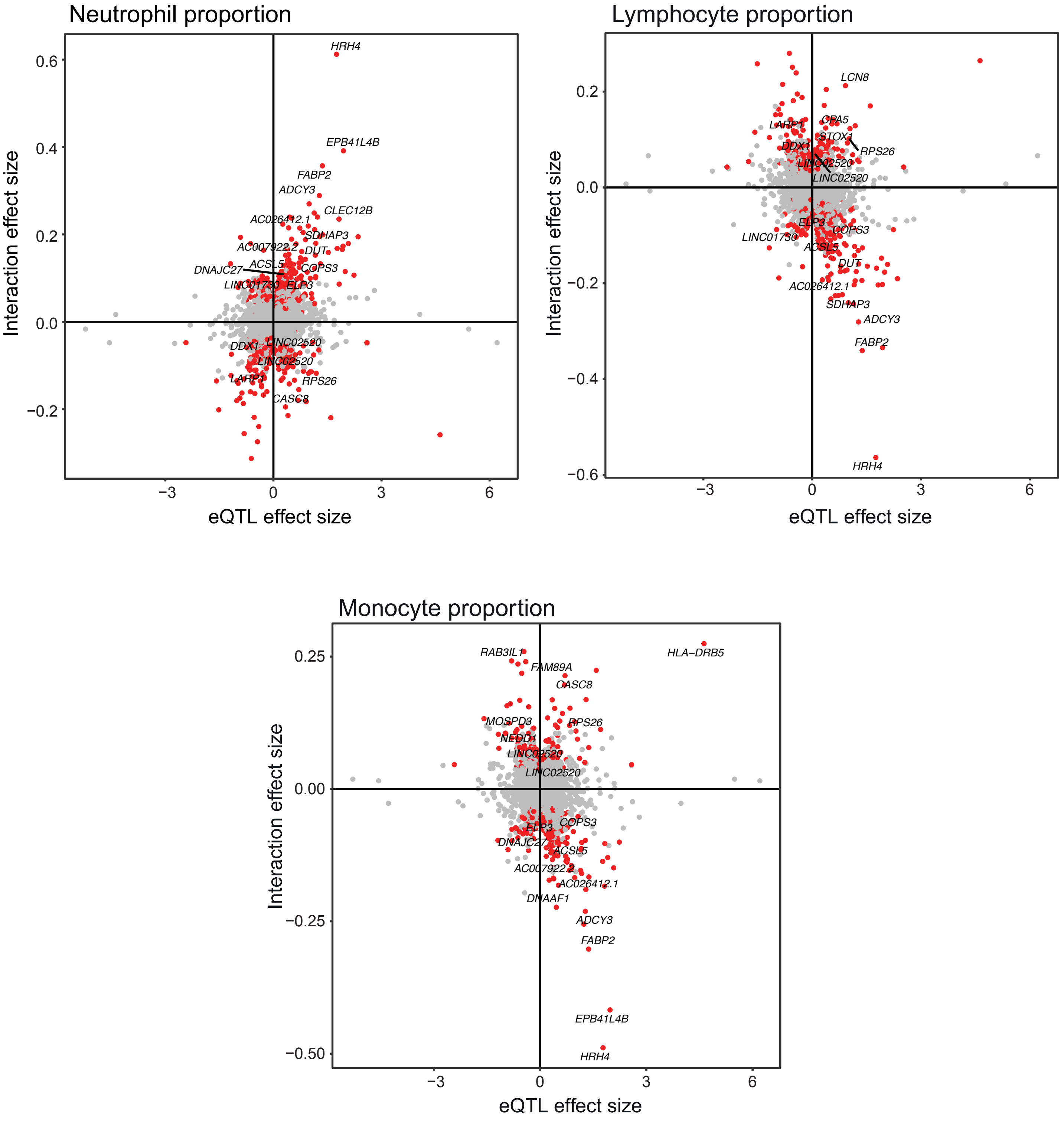

Figure S11: Cell proportion interaction QTL results, related to Figure 2.
eQTL interactions with measured cell proportions. Each point represents an independent eSNP-eGene pair, with the interaction effect size plotted against the genotype effect estimated using a linear mixed model. eQTLs with bigger effects with increased cell proportion are found in the top right and bottom left quadrants. Red colour indicates a significant interaction between genotype and cell proportion (nominal p-values calculated from likelihood ratio test and corrected using Benjamini-Hochberg procedure; significance FDR<0.05), with the most significant results labelled with the eGene name.

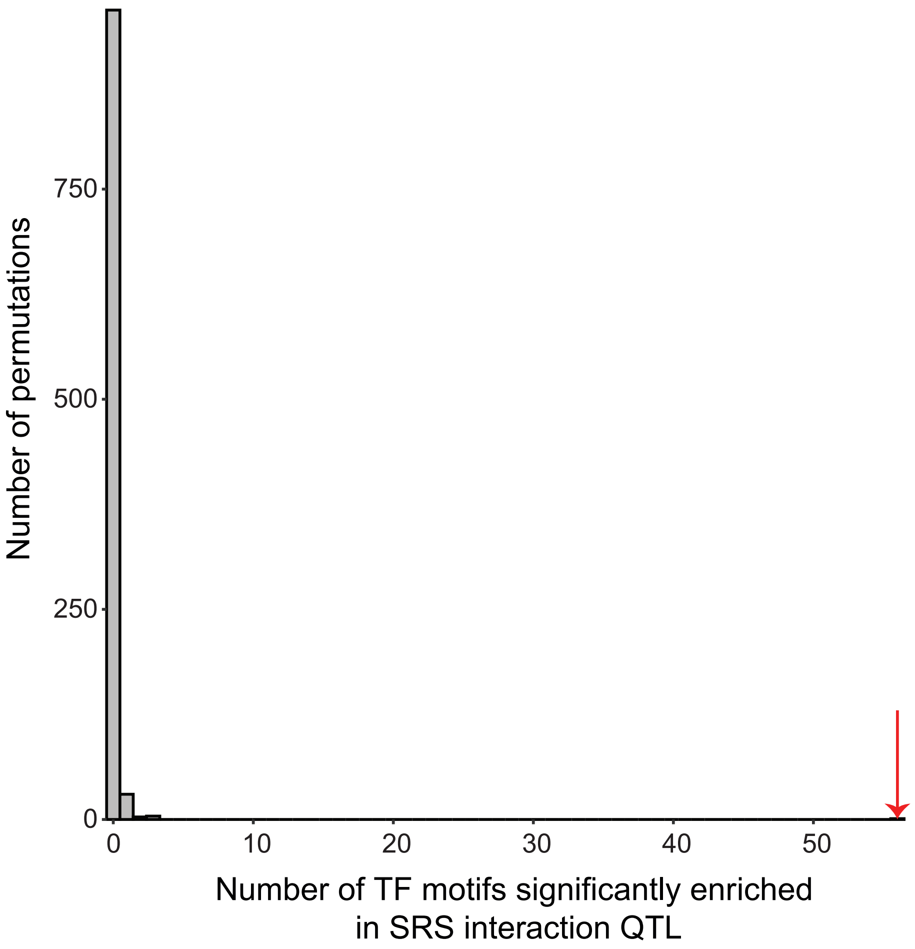

Figure S12: Distribution of transcription factor binding site enrichment results from permutation analysis, related to Figure 3.
eQTL interaction status was permuted across all eQTL signals and the TFBS enrichment tests were repeated, with the number of significantly enriched motifs for each permutation plotted as a histogram. The observed result is marked with a red arrow.

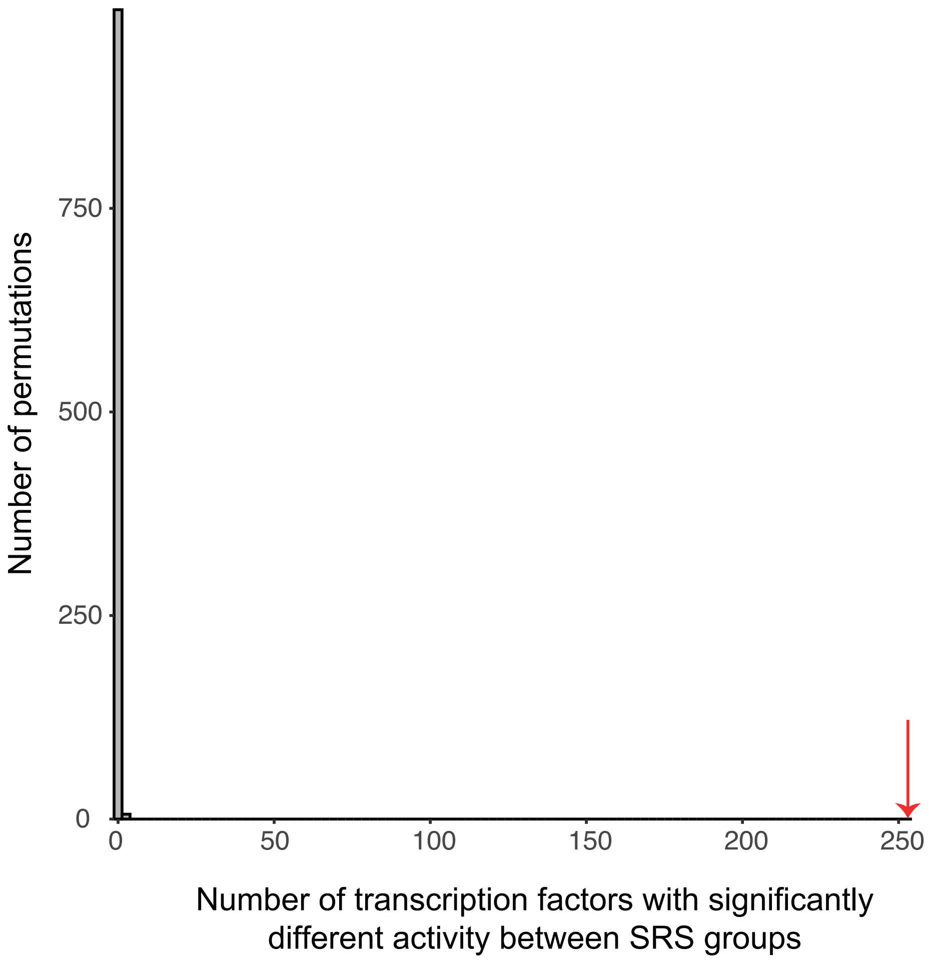

Figure S13: Distribution of DoRothEA inferred TF activity differences from permutation analysis, related to Figure 3.
SRS status was permuted across samples and inferred transcription factor activity compared between groups, with the number of significantly differing TFs for each permutation plotted as a histogram. The observed result is marked with a red arrow.

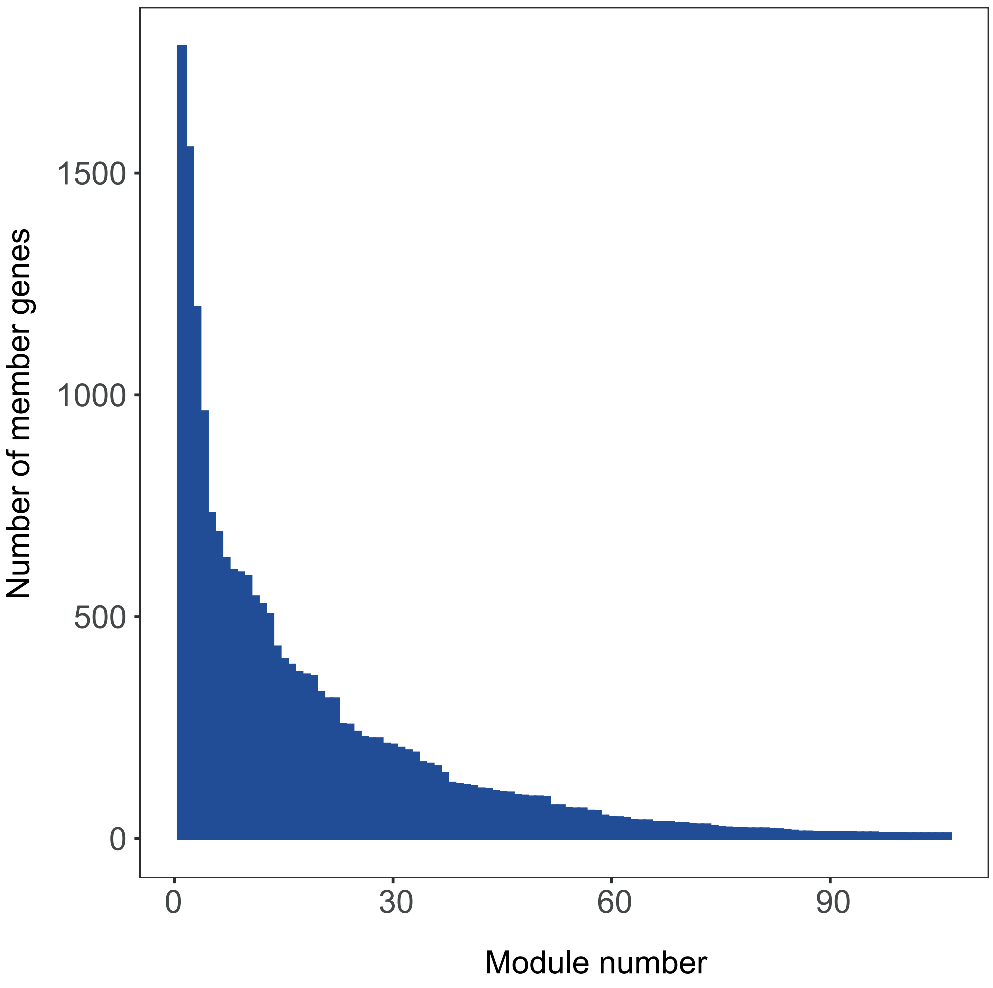

Figure S14: Histogram of co-expression module size, related to Figure 4.
The number of genes in each module ranged from 11 to 1,785.

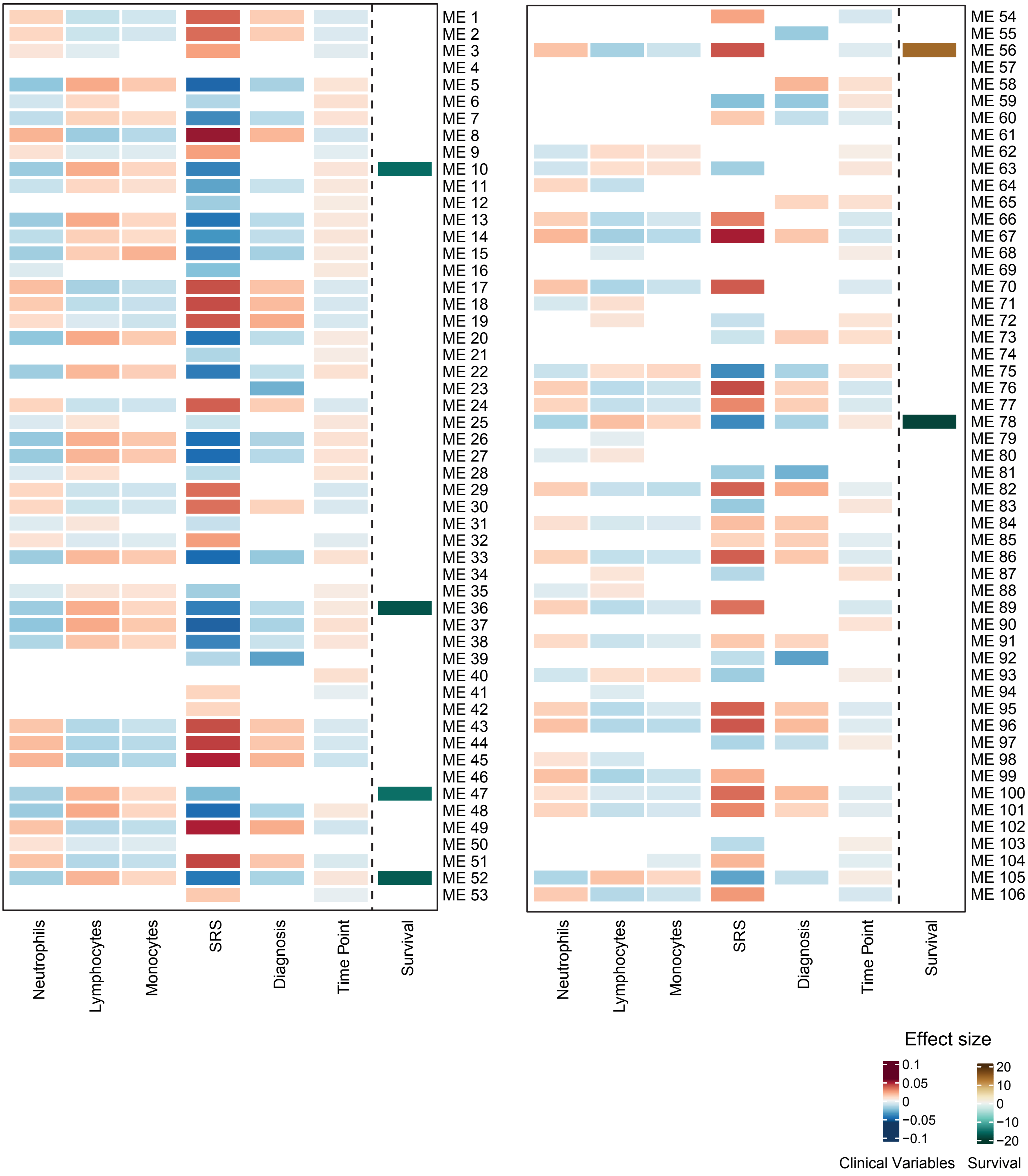

Figure S15: Module eigengene associations with sepsis endophenotypes, related to Figure 4. Heatmap showing significant associations between all module eigengenes (MEs) and clinical phenotypes. MEs were tested for differential expression with measured cell proportions, SRS1 status, diagnosis (CAP or FP) and time point (day 1, 3, 5) using a linear mixed model and likelihood ratio test. Association of each ME with survival up to 28 days was tested using a Cox proportional hazards model. P-values were corrected for multiple testing using the Benjamini-Hochberg procedure.

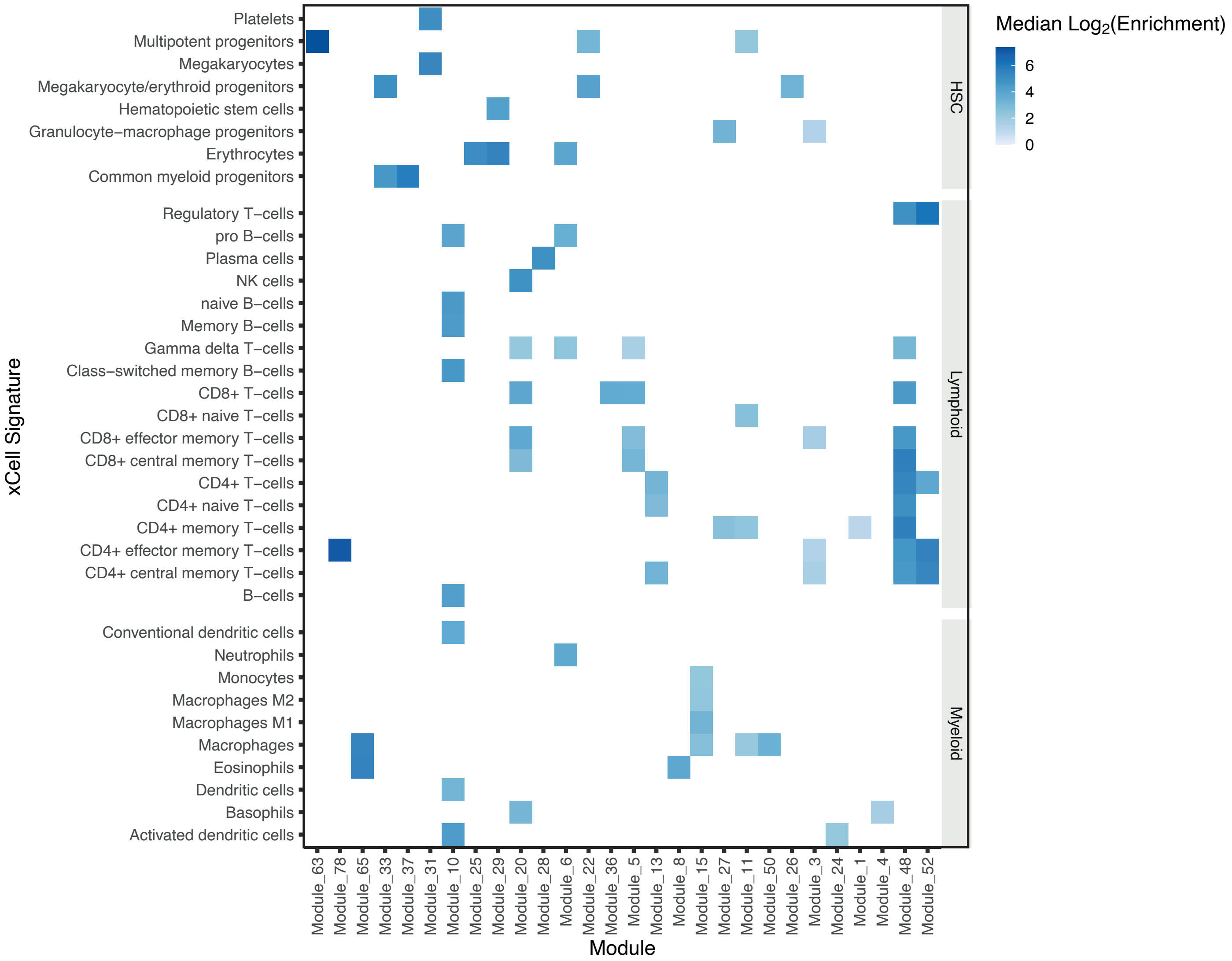

Figure S16: Enrichment of xCell marker genes in module members, related to Figure 4. Modules were tested for enrichment of xCell gene signatures derived from large whole blood transcriptomic studies. Modules shown had significant enrichment for at least one signature. Enrichment of gene signatures was performed using a hypergeometric test using the phyper function in R. The entire set of expressed genes was considered the background for enrichment. P-values were corrected using the Benjamini-Hochberg FDR procedure. Since one cell type often had multiple signatures in xCell, the median odds of enrichment per cell type in xCell were plotted for any signatures that passed a q-value cutoff of 0.05.

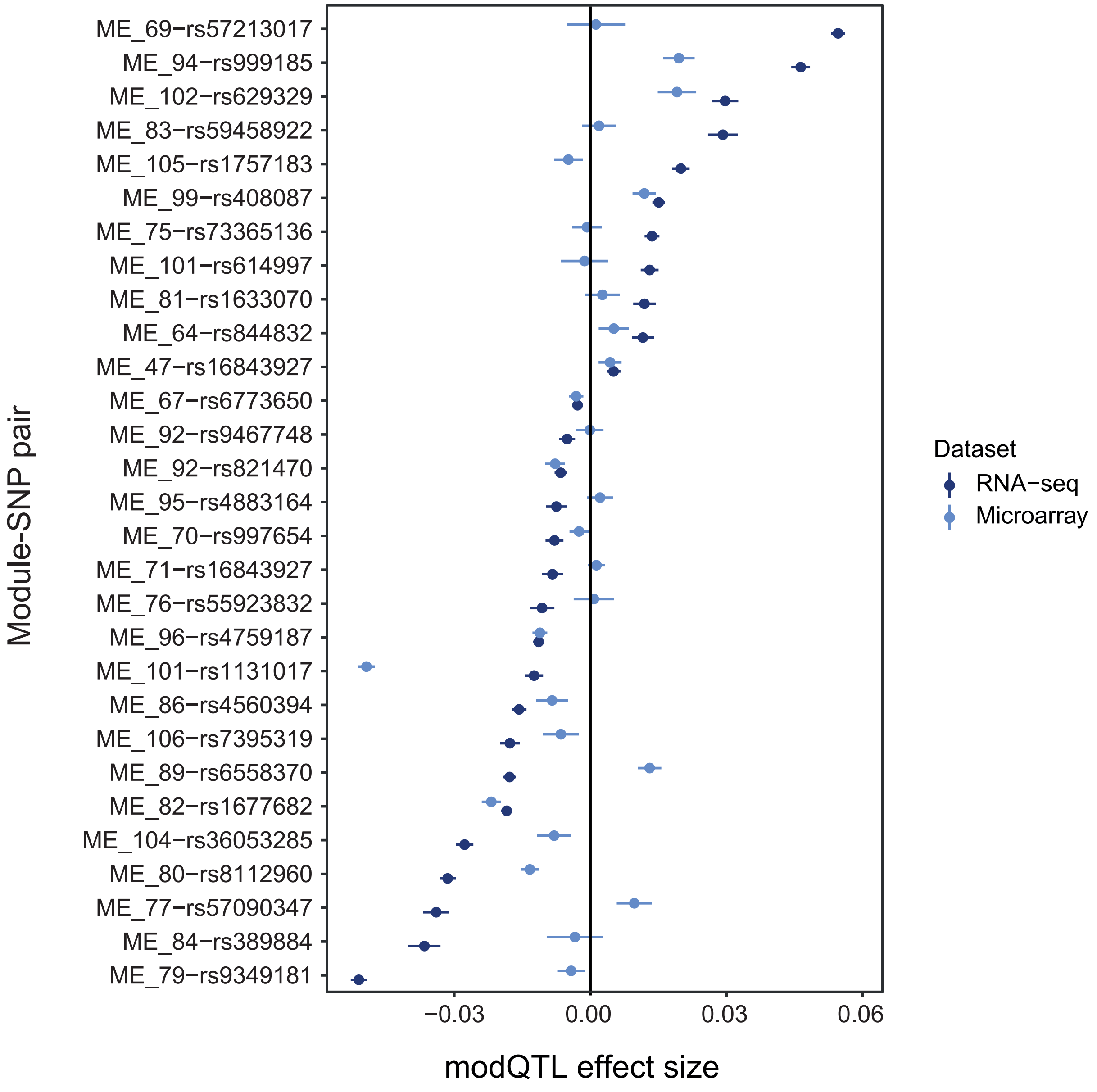

Figure S17: Replication of modQTL effects in a microarray sepsis cohort, related to Figure 4.

Forest plot of modQTL replicated in a validation cohort. Of the 29 modQTL that could be tested, 16 were replicated (nominal p-value from likelihood ratio test <0.05) with consistent direction of effect. Effect sizes from the linear mixed model in the discovery RNA-seq data set and the replication microarray data set are shown as points with 95% confidence intervals as lines.

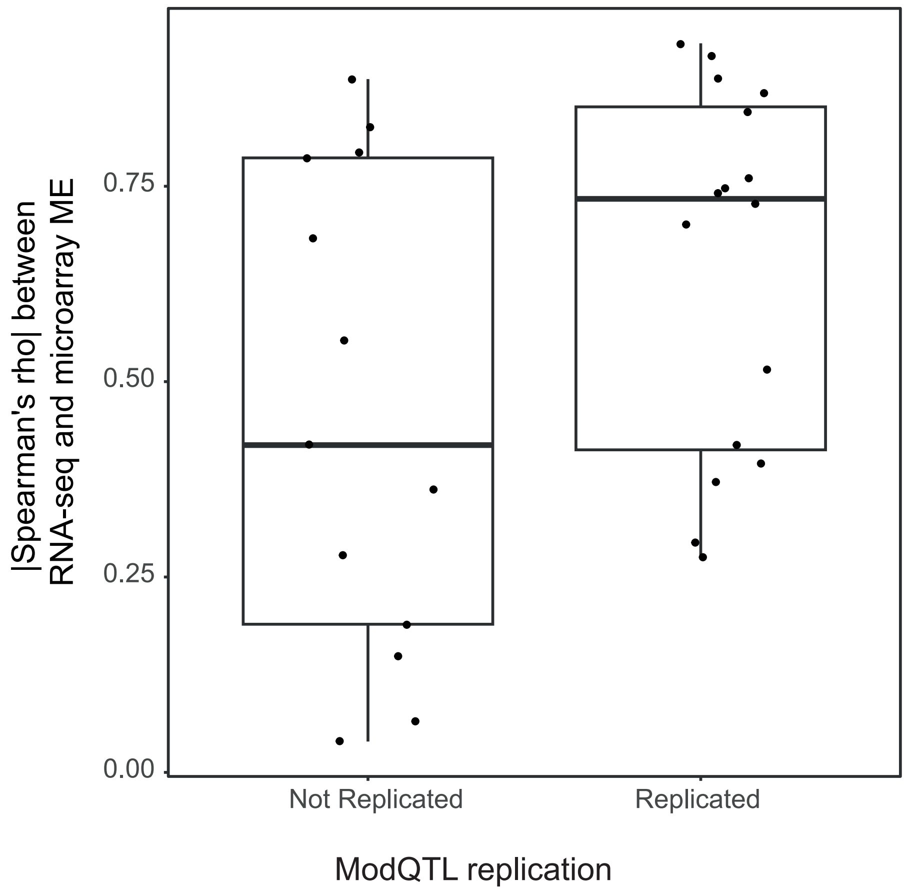

Figure S18: Correlation of module eigengenes across technologies by replication status, related to Figure 4.
Module eigengenes were calculated using the same module gene sets in a microarray cohort with 135 overlapping samples. Similarity between module eigengenes were tested using Spearman's rho for the overlapping samples. Module QTL that replicated (nominal p-value from likelihood ratio test < 0.05, same direction of effect) with the non-overlapping samples had better correlated eigengenes between the two datasets.

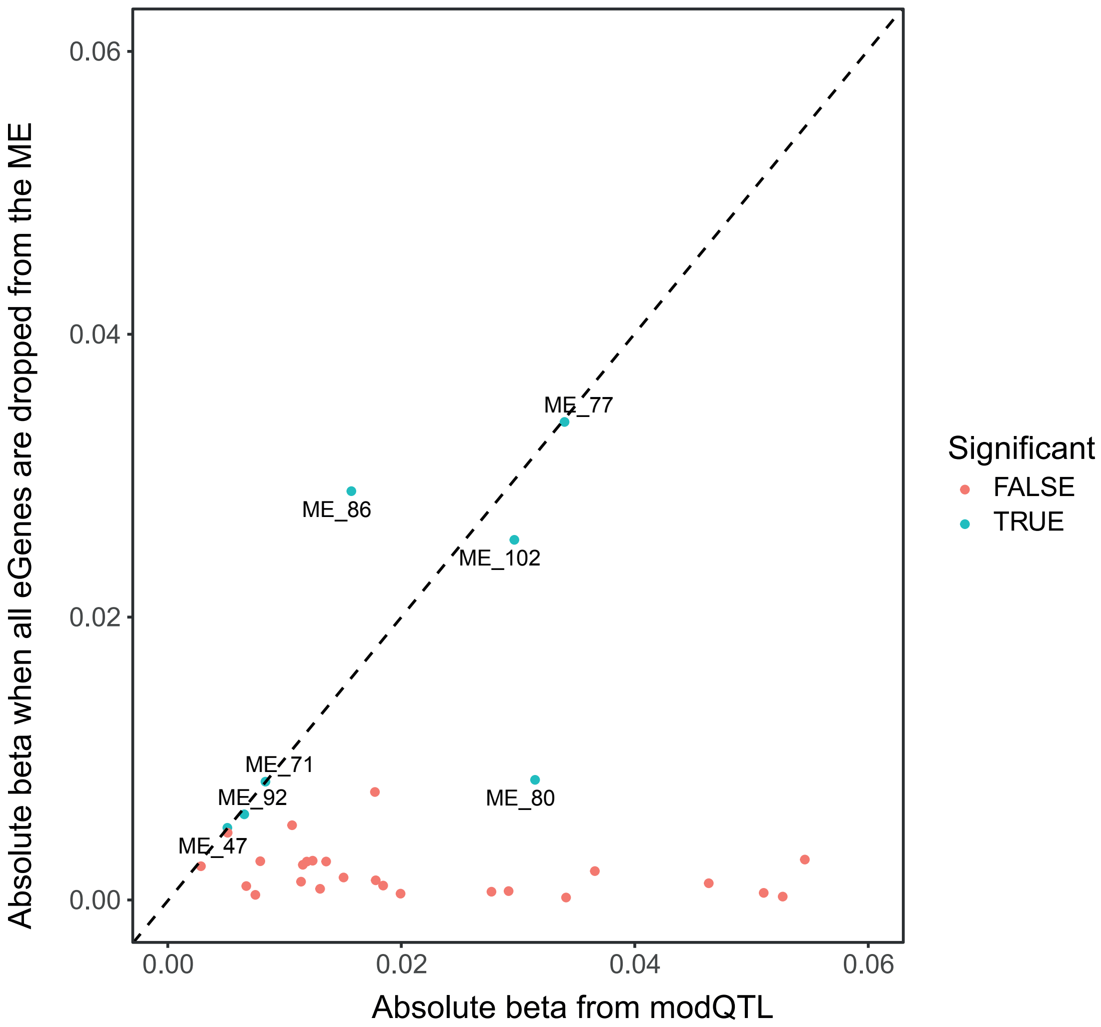

Figure S19: ModQTL sensitivity analysis, related to Figure 4.
Module eigengenes were recalculated excluding any eGenes for the associated modQTL SNPs, and the modQTL retested with a linear mixed model and likelihood ratio test. The new beta value is plotted against the original for the same SNP-ME pair, and coloured by whether the association remained significant.

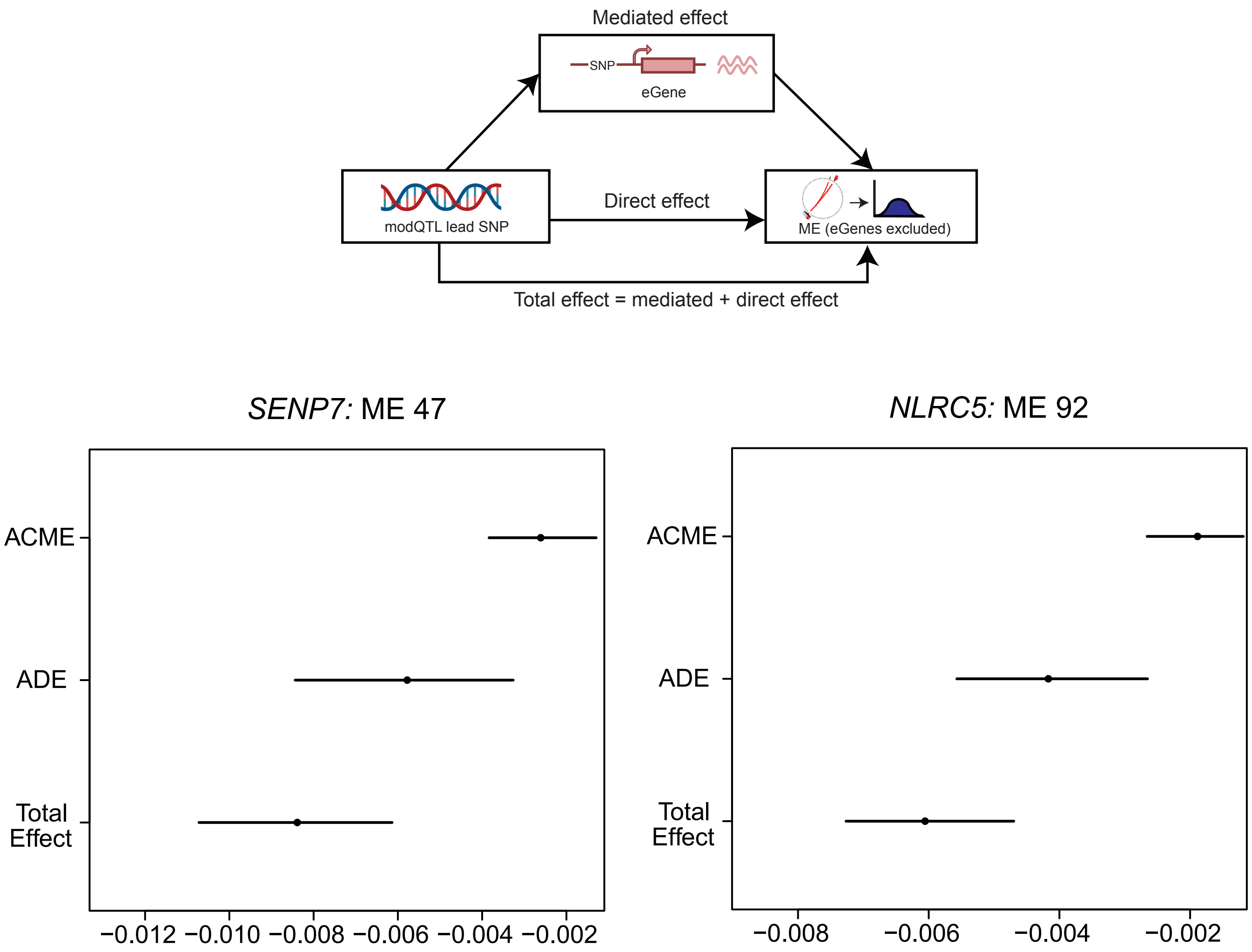

Figure S20: ModQTL mediation results, related to Figure 4.
We tested for mediation of associations between the lead modQTL eSNP and the recalculated eigengene by the modQTL SNPs' target cis-eGene(s). The Average Causal Mediated Effect (ACME), Average Direct Effect (ADE) and total effects are plotted for two modQTL of biological interest.

Supplementary Tables

	Full GWAS cohort (n=997)	SRS1ever (n=440)	SRS1never (n=557)
**SRS1ever: n (%)**	440 (44%)		
**Male sex: n (%)**	544 (55%)	211 (48%)	333 (60%)
**Age: median (range)**	65 (18-92)	65 (18-89)	65 (21-92)
**Source of Sepsis: n CAP (%)**	657 (66%)	229 (52%)	428 (77%)
**Mortality (28 days): n (%)**	159 (16%)	86 (20%)	73 (13%)
**EUR (self-reported): n (%)**	962 (96%)	429 (98%)	533 (96%)

	RNA-seq eQTL cohort (n=638)		
**Male sex: n (%)**	285 (45%)		
**Age: median (range)**	63 (18-90)		
**Source of Sepsis: n CAP (%)**	420 (66%)		
**Mortality (28 days): n (%)**	70 (11%)		
**EUR (self-reported): n (%)**	611 (96%)		
	**Sample level (n=864)**		
	**D1 (n=278)**	**D3 (n=301)**	**D5 (n=244)**
**SRS1: n (%)**	156 (56%)	114 (38%)	59 (24%)
**Neutrophil proportion: median (range)**	0.87 (0.29-0.98)	0.86 (0.18-1.00)	0.83 (0.40-1.00)
**Lymphocyte proportion: median (range)**	0.070 (0-0.46)	0.078 (0-0.35)	0.092 (0-0.49)
**Monocyte proportion: median (range)**	0.050 (0-0.43)	0.050 (0-0.66)	0.064 (0-0.053)

Table S1: Cohort characteristics, related to STAR Methods.
Summary of key demographic and clinical characteristics of the cohorts used in GWAS and eQTL analyses. GWAS: genome-wide association study; SRS: sepsis response signature; CAP: community-acquired pneumonia; EUR: European; eQTL: expression quantitative trait locus; D1: Day 1 of ICU admission; D3: Day 3 of ICU admission; D5: Day 5 of ICU admission.

**Source**	**Estimate**	**Standard Error**
Genetic variance [V(G)]	0.138966	0.069138
Residual variance [Ve]	0.106676	0.067543
Phenotypic variance [Vp]	0.245642	0.011162
V(G)/Vp	0.565726	0.276889
logL	182.189	
logL0	180.055	
LRT	4.269	
df	1	
Pvalue	1.94E-02	
Sample size	997	

**Fixed effects**	**Estimate**	**Standard error**
Population mean	0.084509	0.177485
Age	0.0102	0.00626
Age^2	-0.000081	0.000053
Genotyping PC1	-0.580608	1.090227
Genotyping PC2	0.199891	0.762984
Genotyping PC3	0.167896	0.682518
Genotyping PC4	-0.096222	0.686925
Genotyping PC5	0.209685	0.634264
Genotyping PC6	0.130617	0.64264
Genotyping PC7	-0.280468	0.611016
Sex	0.122251	0.031656

Table S2: Heritability results, related to Figure 1.
Summary of GCTA-GREML analysis showing estimated variance in SRS phenotype explained by common variants by restricted maximum likelihood. logL0: log-likelihood under the null hypothesis that V(G)=0; logL: log-likelihood under the alternative hypothesis that V(G) ≠ 0; LRT: log-likelihood ratio test statistic, $LRT = 2[L(H_1) - L(H_0)]$.

GAinS Investigators

The following GainS investigators, listed alphabetically by institution, were involved in patient recruitment, sample collection, or sample processing:
Jenni Addison[1], Helen Galley[1], Sally Hall[1], Sian Roughton[1], Jane Taylor[1], Heather Tennant[1], Nigel Webster[1], Achyut Guleri[2], Natalia Waddington[2], Dilshan Arawwawala[3], John Durcan[3], Christine Mitchell-Inwang[3], Alasdair Short[3], Susan Smolen[3], Karen Swan[3], Sarah Williams[3], Emily Errington[4], Tony Gordon[4], Maie Templeton[4], Marie McCauley[5], Pyda Venatesh[5], Geraldine Ward[5], Simon Baudouin[6,23], Sally Grier[7], Elaine Hall[7], Charley Higham[6], Jasmeet Soar[7], Stephen Brett[8], David Kitson[8], Juan Moreno[8], Laura Mountford[8], Robert Wilson[8], Peter Hall[9], Jackie Hewlett[9], Stuart McKechnie[10,11], Roser Faras-Arraya[11], Christopher Garrard[11], Paula Hutton[11], Julian Millo[11], Penny Parsons[11], Alex Smiths[11], Duncan Young[11], Parizade Raymode[12], Jasmeet Soar[12], Prem Andreou[13], Sarah Bowrey[13], Dawn Hales[13], Sandra Kazembe[13], Natalie Rich[13], Emma Roberts[13], Jonathan Thompson[13], Simon Fletcher[14], Georgina Glister[14], Melissa Rosbergen[14], Jeronimo Moreno Cuesta[15], Julian Bion[16], Ronald Carrera[16], Sarah Lees[16], Joanne Millar[16], Natalie Mitchell[16], Annette Nilson[16], Elsa Jane Perry[16], Sebastian Ruel[16], Jude Wilde[16], Heather Willis[16], Jane Atkinson[17], Abby Brown[17], Nicola Jacques[17], Atul Kapila[17], Heather Prowse[17], Martin Bland[18], Lynne Bullock[18], Donna Harrison[18], Anton Krige[18], Gary Mills[19,20], John Humphreys[19,20], Kelsey Armitage[19,20], Shond Laha[21], Jacqueline Baldwin[21], Angela Walsh[21], Nicola Doherty[21], Stephen Drage[22], Laura Ortiz-Ruiz de Gordoa[22], Sarah Lowes[22], Charley Higham[23], Helen Walsh[23], Verity Calder[23], Catherine Swan[23], Heather Payne[23], David Higgins[24], Sarah Andrews[24], Sarah Mappleback[24], Charles Hinds[25,32], D Watson[26,27], Eleanor McLees[26,27], Alice Purdy[26,27], Martin Stotz[28], Adaeze Ochelli-Okpue[28], Stephen Bonner[29], Iain Whitehead[29], Keith Hugil[29], Victoria Goodridge[29], Louisa Cawthor[29], Martin Kuper[30], Sheik Pahary[30], Geoffrey Bellingan[31], Richard Marshall[31], Hugh Montgomery[31], Jung Hyun Ryu[31], Georgia Bercades[31], Susan Boluda[31], Andrew Bentley[32], Katie Mccalman[32], Fiona Jefferies[32], Alice Allcock[33], Katie Burnham[33], Emma Davenport[33], Cyndi Geoghegan[33], Julian Knight[33], Narelle Maugeri[33], Yuxin Mi[33], and Jayachandran Radhakrishnan[33].

[1]Aberdeen Royal Infirmary, Aberdeen AB25 2ZN, UK.
[2]Blackpool Victoria Hospital, Blackpool FY3 8NR, UK.
[3]Broomfield Hospital, Chelmsford CM1 7ET, UK.
[4]Charing Cross Hospital, London W6 8RF, UK.
[5]Coventry and Warwickshire University Hospital, Coventry CV2 2DX, UK.
[6]Freeman Hospital, Newcastle upon Tyne NE7 7DN, UK.
[7]Frenchay Hospital, Bristol, UK and Southmead Hospital, Bristol BS16 1JE, UK.
[8]Hammersmith Hospital, London W12 0HS, UK.
[9]Huddersfield Royal Infirmary, Huddersfield HD3 3EA, UK.
[10]Oxford University Hospitals NHS Foundation Trust, Oxford
[11]John Radcliffe Hospital, Headington, Oxford OX3 9DU, UK.
[12]Kettering General Hospital, Kettering NN16 8UZ, UK.
[13]Leicester Royal Infirmary, Leicester LE1 5WW, UK.
[14]Norfolk and Norwich University Hospital, Norwich NR4 7UY, UK.
[15]North Middlesex Hospital, London N18 1QX, UK.
[16]Queen Elizabeth Hospital, Birmingham B15 2GW, UK.
[17]Royal Berkshire Hospital, Reading RG1 5AN, UK.
[18]Royal Blackburn Hospital, Blackburn BB2 3HH, UK.

[19]Royal Hallamshire Hospital, Sheffield S10 2JF, UK.
[20]Northern General Hospital, Sheffield S5 7AU, UK.
[21]Royal Preston Hospital, Preston PR2 9HT, UK.
[22]Royal Sussex County Hospital, Brighton BN2 5BE, UK.
[23]Royal Victoria Infirmary, Newcastle upon Tyne NE1 4LP, UK.
[24]Southend Hospital, Westcliff-on-Sea SS0 0RY, UK.
[25]Centre for Translational Medicine & Therapeutics, William Harvey Research Institute, Faculty of Medicine & Dentistry, Queen Mary University of London, London, UK
[26]St Bartholomew's Hospital, London EC1A 7BE, UK.
[27]Royal London Hospital, London E1 1FR, UK.
[28]St Mary's Hospital, London W2 1NY, UK.
[29]James Cook University Hospital, Middlesbrough TS4 3BW, UK.
[30]Whittington Hospital, London N19 5NF, UK.
[31]University College London Hospital, UCLH, London NW1 2BU, UK.
[32]Wythenshawe Hospital, Manchester M23 9LT, UK.
[33]Centre for Human Genetics, University of Oxford, Oxford, UK
